# The Association Between Self-Reported Screen Time, Social Media Addiction, and Sleep Among Norwegian University Students

**DOI:** 10.3389/fpubh.2021.794307

**Published:** 2021-12-16

**Authors:** Gunnhild J. Hjetland, Jens C. Skogen, Mari Hysing, Børge Sivertsen

**Affiliations:** ^1^Department of Health Promotion, Norwegian Institute of Public Health, Bergen, Norway; ^2^Alcohol and Drug Research Western Norway, Stavanger University Hospital, Stavanger, Norway; ^3^Centre for Evaluation of Public Health Measures, Norwegian Institute of Public Health, Oslo, Norway; ^4^Department of Psychosocial Science, University of Bergen, Bergen, Norway; ^5^Department of Research and Innovation, Helse Fonna HF, Haugesund, Norway; ^6^Department of Mental Health, Norwegian University of Science and Technology, Trondheim, Norway

**Keywords:** sleep, insomnia, screen time, social media addiction, students

## Abstract

The aim of this study was to assess the relationship between daily screen time and sleep, evening screen time and sleep, and between social media addiction and sleep in a student population. This cross-sectional study is based on data from a national survey of all college and university students in Norway (the SHoT2018 study; *n* = 49,051). The sleep outcomes were sleep duration, sleep onset latency, sleep efficiency, and insomnia operationalized according to formal DSM-5 criteria. The results show a strong negative association between time spent on screen-based devices and sleep quality and quantity, and where screen use in bed had more consistent negative associations with sleep. Furthermore, there were higher rates of insomnia among those with higher levels of addiction, and curvilinear relationships with sleep duration, sleep onset latency, and sleep efficiency. Those with higher levels of addiction also had more evening screen time. The findings suggest that screen use plays an important role in students' sleep quantity and quality, where evening screen time has a stronger relationship with sleep compared to total daily screen time. The results also suggest a role of social media addiction, and addictive social media use may be a target for intervention in order to reduce evening screen time.

## Introduction

Sleep problems are prevalent among students (1, 2). A recent large-scale study of over 7,000 American university students found that only one third slept for more than 7 h each night (1), falling short of the recommended 7–9 h (3). In a recent national study of Norwegian college and university students, the prevalence of insomnia was found to be 34.2 % among female students and 22.2 % among male students (4). Poor sleep has detrimental effects on physical (5–7) and mental health (8, 9), and poor sleep quality and daytime sleepiness can impair students' academic performance (10–12).

Screen-based devices have permeated society and become a natural part of many peoples' everyday lives. Young people are particularly frequent users of screen-based devices (13), including activities such as video gaming, watching movies and TV series, and using social media. The extensive use of screen-based technology has been linked to poor sleep, where daily screen time is negatively associated with sleep quantity and quality (14). This association may be driven by screen use around bedtime (13), which is common (15, 16). A study of Norwegian students found that 76 % of the respondents used their mobile phone after going to bed, while only 5 % reported that they never used screen-based devices in bed (15). While the use of screens during the day may be hard to limit due to school or work obligations, pre-sleep screen use may in many cases be a highly modifiable activity with the potential to significantly improve sleep.

Evening screen time can impair sleep through several mechanisms, as proposed by Cain and Gradisar (17). First, screen time may replace time spent asleep in the evening, delaying sleep onset (16, 18). Second, electronic screens emit short-wavelength light that can increase alertness and delay the circadian rhythm through suppressed melatonin secretion, which would normally occur in the evening (19). Illustrating this point, Grønli et al. (20) found that reading a story from a tablet at bedtime reduced subjective sleepiness and delayed and reduced slow wave activity after sleep onset, compared to reading the same story from a book. Third, screen-based activities in the evening may cause cognitive and affective arousal and subsequent difficulties falling asleep (17, 18).

Compared to watching television or other “passive” screen-based activities, social media may be particularly potent in terms of replacing sleep and pre-bedtime arousal (18, 21). Social media are “highly interactive platforms *via* which individuals and communities share, co-create, discuss, and modify user-generated content” (22). Some individuals develop social media addiction, i.e., an excessive use of social media that interferes with other activities, relationships, and obligations of daily life (23). Social media addiction has been associated with more screen use during the evening and night (24, 25), and with poorer subjective sleep quality among students and adults (25–27). Social media addiction as a concept has been debated in the literature (28). For the purpose of this study, we use the term “social media addiction,” in line with other research (29). However, we appreciate that social media addiction is not a disorder recognized in the Diagnostic and Statistical Manual of Mental Disorders (DSM-5) or the International Classification of Diseases 11th Revision (ICD-11).

The majority of studies on screen time and sleep have focused on children or adolescents (30), although a few studies have confirmed an association between screen time and sleep among young adults and students (14, 15, 24). Further, the majority of studies assess the relationship between screen use and sleep duration, and to a lesser extent other sleep outcomes such as sleep onset latency, sleep efficiency, and sleep problems (30). The aim of the present study was to investigate the relationship between total and evening screen time and sleep in a student population, assessing both sleep quantity and quality. In addition, we included a proxy for the DSM-5 criteria for insomnia. We also aimed to investigate the association of social media addiction with both evening screen time and with sleep outcomes. In line with the recommendations by Hale & Guan (30), we adjusted for other factors associated with both screen time and sleep, such as gender and age (31, 32), and with obesity and physical inactivity (33–36). Furthermore, we adjusted for the student-specific factors study program and number of fail grades.

Our hypotheses were that: (1) Total screen time, evening screen time, and social media addiction are all negatively associated with sleep duration and sleep efficiency, and positively associated with sleep onset latency and insomnia; (2) social media addiction is positively associated with evening screen time, and that (3) evening screen time is more strongly associated with sleep parameters than total screen time.

## Materials and Methods

### Procedure

The present study was based on data from the SHoT2018 study; a national survey of all college and university students in Norway. The SHoT2018 study was conducted from February to April 2018, and all fulltime Norwegian college and university students between the ages of 18 and 35 were invited. Of the 162,412 students who fulfilled the inclusion criteria, 50,054 provided valid responses to the web-based questionnaires, resulting in a response rate 30.8 %. Detailed information about the study can be found elsewhere (37).

### Ethics

The SHoT2018 study was approved by the Regional Committee for Medical and Health Ethics in Western Norway (No. 2017/1176). Potential participants received a detailed introduction to the study, after which they provided an electronic informed consent.

### Instruments

#### Use of Electronic Devices at Bedtime

Few well-validated questionnaires assessing screen time and the use of modern electronic devices are available. Consequently, we chose to include an instrument assessing the use of a wide range of electronic devices, following a thorough review of the literature. More details can be found elsewhere (38). The participants were asked to indicate which electronic devices they used after going to bed in the evening: Television, PC/Mac, tablet, mobile phone, radio/music player, and gaming console. Next, participants were asked what they were using the electronic devices for: Watching movies/TV series, checking social media, surfing the internet, listening to music/audiobooks, games/gaming, and reading study-related material. The participants could indicate as many devices and activities as they deemed relevant. Those who indicated that they used one or more electronic devices after going to bed were asked about the number of evenings they did so over the course of 1 week (1–7 evenings), and how much time they spent using these devices each evening. Evening screen time was indicated using a drop-down menu ranging from 5 min to “more than 6 h,” with increasing intervals from 5 min to 1 h. For the purpose of this study, total evening screen time was calculated by multiplying evening screen time with the number of evenings using electronic devices, divided by seven to estimate a daily average. In addition to using these variables continuously, we also divided responses into 5 categories, according to percentiles.

#### Social Media Addiction

Self-perceived social media addiction was indicated by the question: “I perceive myself as addicted to social media.” Participants indicated how well this statement pertained to them on a 5-point Likert scale ranging from “not at all” to “very much.”

#### Total Screen Time

The participants were asked to estimate how much time they spent on screen-based activities across 24 h using a drop-down menu ranging from 0 to 18 or more with 1-h intervals.

#### Sleep Variables

For the purpose of this study, three sleep outcomes measuring quantity and quality of sleep were used, indicated separately for weekdays and weekends. Sleep duration was calculated as the reported time in bed (TIB) minus sleep onset latency (SOL) and wake-after-sleep-onset. Sleep efficiency (SE) was calculated as sleep duration divided by TIB multiplied by 100.

Insomnia was assessed by items in line with the DSM-5 criteria for insomnia and participants were classified as having insomnia if they indicated that they had: (a) experienced either difficulties initiating sleep, difficulties maintaining sleep, or early morning awakenings, at least three nights per week; (b) experienced daytime sleepiness or tiredness for at least 3 days a week; and (c) these sleep problems had lasted for at least 3 months. More details about the included sleep inventory has been published elsewhere (4, 32).

### Confounders

#### Age and Gender

Students provided their age and gender (“male,” “female,” or “non-binary”).

#### Physical Exercise

The frequency of physical exercise was assessed using the following preamble: “With physical exercise we mean that you for example go for a walk, go skiing, swim or take part in a sport,” followed by the question “How frequently do you perform physical exercise?” The following response options were available: Never, Less than once a week, Once a week, 2–3 times per week, Almost every day. As an index of physical activity vs physical inactivity, a variable was dichotomized using 2 times per week as the cut-off value (39).

#### Body Mass Index

Body mass index (BMI) was calculated using self-reported body weight divided by the squared height $\left(\frac{kg}{m^{2}}\right)$. We then divided the index into four groups, using the following criteria: underweight (BMI <18.5), normal weight (BMI: 18.5–24.9), overweight (BMI: 25.0–29.9), and obesity (BMI ≥ 30) (40).

#### Study Program

The participants indicated whether they attended a one-year program, short professional studies, long professional studies, a master's program (2 years or less), or “other.”

#### Number of Fail Grades

The number of fail grades was assessed using the following question: “Have you failed any exams since you started college/university?” with the response options “yes” and “no.” Those confirming that they had failed any exams indicated the number of failed exams using a drop-down menu ranging from one to 10 or more.

### Handling of Invalid and Missing Information

A total of 50,054 students participated in the SHOT2018-survey. For the sleep variables, *n* = 111 observations were removed as they had invalid responses, including (a) SOL or WASO >12 h, (b) SOL + WASO > TIB, (c) negative values of sleep duration and sleep efficiency. Furthermore, *n* = 113 observations were removed for the purposes of this study as they indicated a non-binary gender, and the group were deemed too small be meaningfully presented separately. Those with missing information about gender (*n* = 102) and age (*n* = 677) were also removed. The sample size eligible for the present study was *N* = 49,051 (97.9 % of the original sample).

### Statistical Analyses

The mean and 95 % confidence intervals (CI95%) of the continuous variables (total screen time, total evening screen time, sleep duration, sleep onset latency, sleep efficiency), the median and interquartile range of social media addiction, and the proportion with corresponding 95 % confidence intervals of insomnia across gender is presented in Table 1. For the main analyses, the continuous dependent variables (weekday sleep duration, sleep onset latency, and sleep efficiency) linear regression models were computed, and logistic regression models were employed for insomnia. Percentile variables approximating quintiles were constructed for total digital use and total digital use evening, while the full set of response categories (from “not at all” to “very much”) of social media addiction were entered into separate regression models as indicator variables. Post-estimations were used to produce the estimated mean (for continuous dependent variables) and proportions (for insomnia) with corresponding CI95% across levels of the independent variables. For each independent-dependent combination, pairwise adjacent comparisons were performed across factor levels of the independent variable. In order to maximize the number of observations, pairwise-deletion was used for each independent-dependent variable dyad, with observations ranging from 46,999 (social media addiction and insomnia) to 40,171 (total sleep duration and total digital use evenings). The main analyses are presented as graphs in **Figures 2**–**5**.

**Table 1:** Description of study variables across gender.

	***N***	***N*_males_**	***N*_females_**	**Male**	**Female**	***P*-value**
**Independent variables—digital use**						
Total screen time^a^, daily average [mean (CI95%)]	48,184	14,884	33,300	7:54 (7:51–7:58)	6:58 (6:56–7:01)	<0.001^b^
Total evening screen time^a^, daily average [mean (CI95%)]	42,743	12,783	29,960	0:46 (0:46–0:47)	0:46 (0:45–0:47)	= 0.951^b^
Social media addiction [median (IQR)]	48,583	14,927	33,656	2 (2–3)	3 (2–4)	<0.001^c^
**Dependent variables—sleep**						
Total sleep time^a^ [mean (CI95%)]	47,241	14,550	32,691	7:25 (7:23–7:26)	7:25 (7:25–7:26)	= 0.256^b^
Sleep onset latency^a^ [mean (CI95%)]	48,675	14,936	33,739	0:44 (0:43–0:44)	0:50 (0:50–0:51)	<0.001^b^
Sleep efficiency [mean (CI95%)]	47,241	14,550	32,691	88.62 (88.43–88.81)	86.93 (86.80–87.06)	<0.001^b^
Insomnia [proportion (CI95%)]	49,051	15,076	33,975	22.1% (21.4–22.7)	34.2% (33.7–34.7)	<0.001^d^

a *Numbers presented in hrs:minutes*.

b *Two-sample t-test with equal variances*.

c *Two-sample Wilcoxon rank-sum (Mann-Whitney) test*.

d *Two-sample test of proportions*.

*CI95%, 95% confidence intervals*.

*IQR, Interquartile range*.

## Results

**Figure 1:**
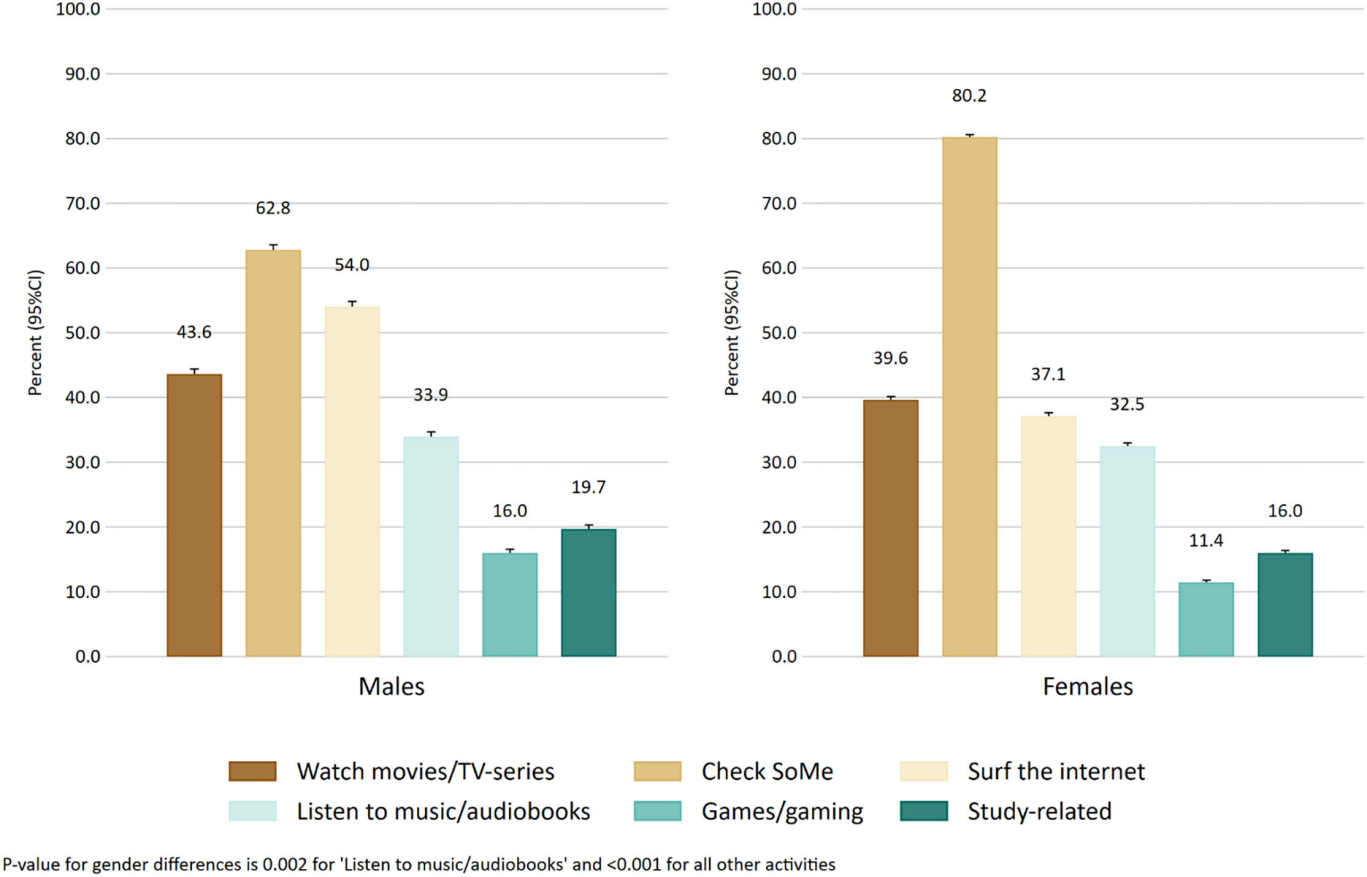
The prevalence of screen-based behaviors in the evening across males and females.

The median age of the sample was 22 years (interquartile range 20–24 years) and 69.3 % were female (Table 1). Males had significantly longer mean total screen time, lower social media addiction, shorter sleep onset latency, higher sleep efficiency, and a lower proportion of insomnia compared to females. There were no sex differences in evening screen time or total sleep time. Figure 1 shows the self-reported proportion of different screen-based activities at bedtime, stratified for males and females. The most commonly reported activity at bedtime was “check social media” for both males and females, followed by “surf the internet” for males and “watch movies/TV-series” for females. Males were more likely to report all activities at bedtime compared to females, except for “check social media,” where males were less likely to report this activity. Table 2 shows the mean and median total screen time and evening screen time for each percentile group.

**Table 2:** Mean, standard deviation, and median across percentile grouping for total screen time and total evening screen time.

		**Total screen time in hours (daily average)^a^**			**Total evening screen time in hours (daily average)^a^**	
**Percentile groups**	***N***	**Mean (SD)**	**Median**	***N***	**Mean (SD)**	**Median**
0–19.9th	11,067	3:14 (0:51)	3:00	9,903	0:07 (0:03)	0:07
20–39.9th	12,350	5:28 (0:30)	5:00	7,210	0:14 (0:02)	0:15
40–59.9th	9,461	7:37 (0:29)	8:00	10,817	0:27 (0:04)	0:30
60–79.9th	8,383	9:46 (0:25)	10:00	8,295	0:50 (0:10)	0:52
80–100th	6,923	13:20 (2:02)	12:00	6,518	2:49 (1:32)	2:00

a *Numbers presented in hrs:minutes*.

*SD, Standard deviation*.

### Social Media Addiction and Evening Screen Time

For different levels of social media addiction, those indicating “not at all” and “not much” had the shortest median evening screen times of 22 min (Table 3). The “some” group had significantly longer median evening screen time of 26 min (*p* < 0.001), and the “much” group of 30 min (*p* < 0.001). The “very much” group had the longest median evening screen time of 34 min (*p* < 0.001).

**Table 3:** Mean, standard deviation, and median of total evening screen time in hours (daily average) across levels of self-reported social media addiction.

	**Total evening screen time in hours (daily average)^a^**		***P*-value^a^**	***P*-value^b^**
**Self-reported SOME-addiction**	**Mean (SD)**	**Median (IQR)**		
Not at all	0:42 (1:02)	0:22 (0:10–0:45)	Ref	N/A
Not much	0:38 (0:57)	0:22 (0:10–0:43)	0.093	0.093
Some	0:44 (1:02)	0:26 (0:11–0:45)	<0.001	<0.001
Much	0:54 (1:12)	0:30 (0:15–1:00)	<0.001	<0.001
Very much	1:08 (1:24)	0:34 (0:17–1:00)	<0.001	<0.001

a *Numbers presented in hrs:minutes*.

*SD, Standard deviation*.

*Overall Kruskal-Wallis equality-populations-rank test, p = 0.0001*.

a *P-value for pairwise comparison with “Not at all” using Kruskal-Wallis equality-of-populations-rank test*.

b *P-value for adjacent pairwise comparisons using Kruskal-Wallis equality-of-populations-rank test*.

### Screen Time and Sleep Duration

**Figure 2:**
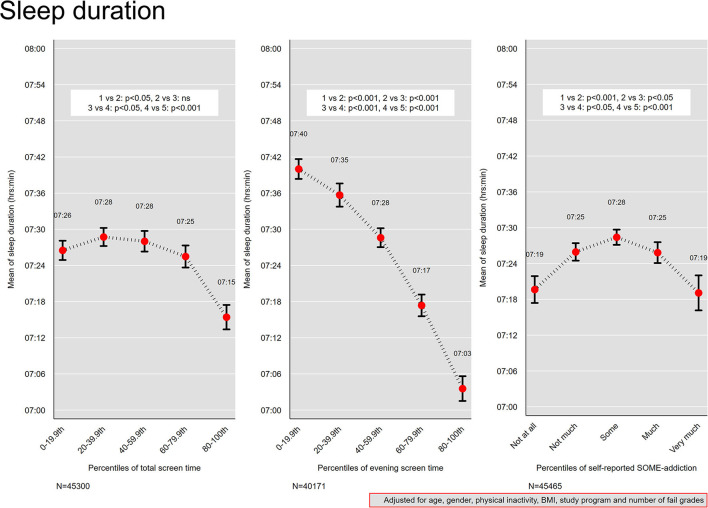
Sleep duration. Mean sleep duration across quintiles of total screen time and evening screen time, and levels of self-reported social media addiction. Results from linear regression models adjusted for age, gender, physical activity, and body mass index. Bars denote 95 % confidence intervals.

In Figure 2, we present the association between screen time and sleep duration adjusted for age, gender, physical activity and body mass index. As there were minimal differences between the fully adjusted associations and associations adjusted for age and gender, we only present the fully adjusted associations. For total screen time, there was a decrease from a mean sleep time of 7 h 28 min in the 40–59th percentile group (median screen time 8 h) to 7 h 26 min in the 60–79th percentile group (median screen time of 10 h), and further to 7 h 12 min in the 80–100th percentile group (median screen time of 12 h). For evening screen time, there was a monotonic association with decreased sleep duration across each percentile group, with a mean sleep time of 7 h 40 min in the 0–19th percentile group (median evening screen time of 7 min) and a mean sleep time of 7 h 3 min in the 80–100th percentile group (median evening screen time of 2 h).

### Social Media Addiction and Sleep Duration

For levels of SOME-addiction, an inverse U-shaped curve was observed (Figure 2), with those reporting “some” addiction reporting the longest sleep duration (mean 7 h 28 min), and those at the extremes (“not at all” and “very much”) reporting the shortest sleep duration (mean sleep time of 7 h 19 min for both).

### Screen Time and Sleep Onset Latency

**Figure 3:**
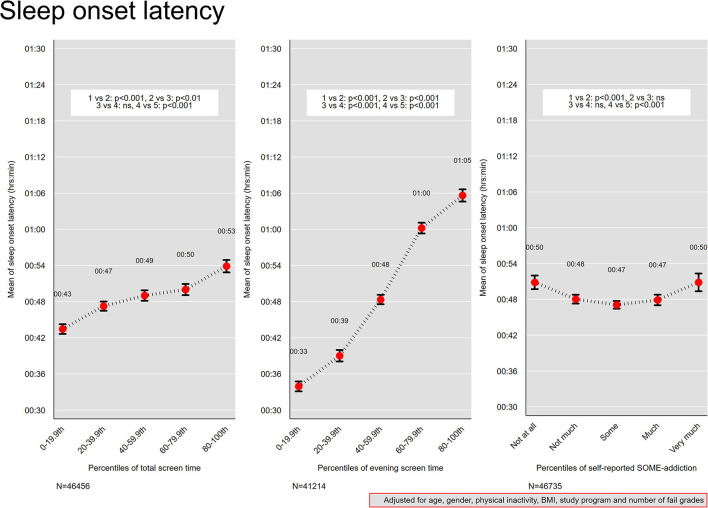
Sleep onset latency. Mean sleep onset latency across quintiles of total screen time and evening screen time, and levels of self-reported social media addiction. Results from linear regression models adjusted for age, gender, physical activity, and body mass index. Bars denote 95 % confidence intervals.

Figure 3 shows the association between screen time and sleep onset latency adjusted for age, gender, physical inactivity, body mass index, study program, and number of fail grades. For percentiles of total screen time and evening screen time, monotonic positive associations were observed, where those in the 0–19th percentile groups of total screen time (median total screen time 3 h) and evening screen time (median evening screen time 7 min) also reported the shortest sleep onset latencies of 43 and 33 min respectively. In comparison, the 80–100th percentile group of total screen time (median screen time 12 h), had a mean sleep onset latency of 53 min. The 80–100th percentile group of evening screen time (median evening screen time 2 h), had a sleep onset latency of 1 h 5 min. The association with sleep onset latency was more marked at the extremes of the total screen time percentiles groups compared with those in 20th−79.9th groups.

### Social Media Addiction and Sleep Onset Latency

For levels of SOME-addiction, a U-shaped curve was observed (Figure 3), with those reporting “some” social media addiction reporting the shortest sleep onset latency (mean of 47 min), and those at the extremes (“not at all” and “very much”) reporting the longest sleep onset latency (mean of 50 min for both).

### Screen Time and Sleep Efficiency

**Figure 4:**
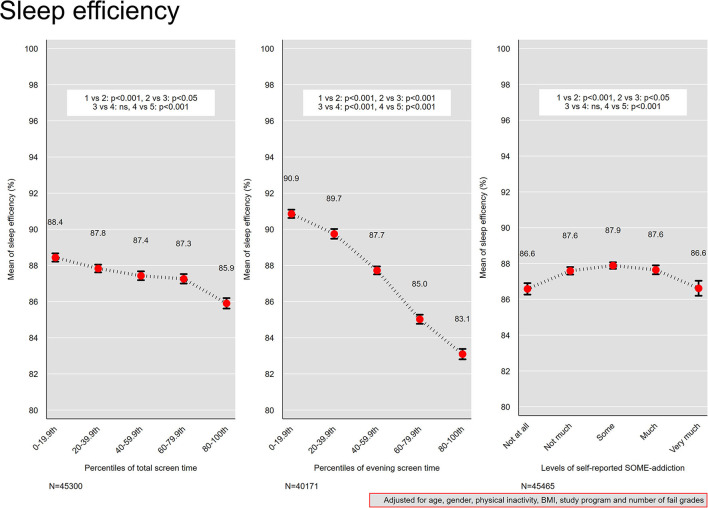
Sleep efficiency. Mean sleep efficiency across quintiles of total screen time and evening screen time, and levels of self-reported social media addiction. Results from linear regression models adjusted for age, gender, physical activity, and body mass index. Bars denote 95 % confidence intervals.

The association between screen time and sleep efficiency is presented in Figure 4. For total screen time, the 0–19.9th percentile group (median total screen time 3 h) had the highest sleep efficiency of 88 %. At the other percentile end, the 80–100th group (total screen time 12 h) had the lowest sleep efficiency of 86 %. A monotonic negative association was observed between evening screen time and sleep efficiency, from 91 % in the 0–19th percentile group (median evening screen time 7 min) to 83 % in the 80–100th percentile group (evening screen time 2 h).

### Social Media Addiction and Sleep Efficiency

An inverse U-shaped curve was observed for self-reported social media addiction and sleep efficiency (Figure 4), with a maximum mean sleep efficiency of 88 % for “some” addiction, and 87 % for “not at all” and “very much.”

### Screen Time and Insomnia

**Figure 5:**
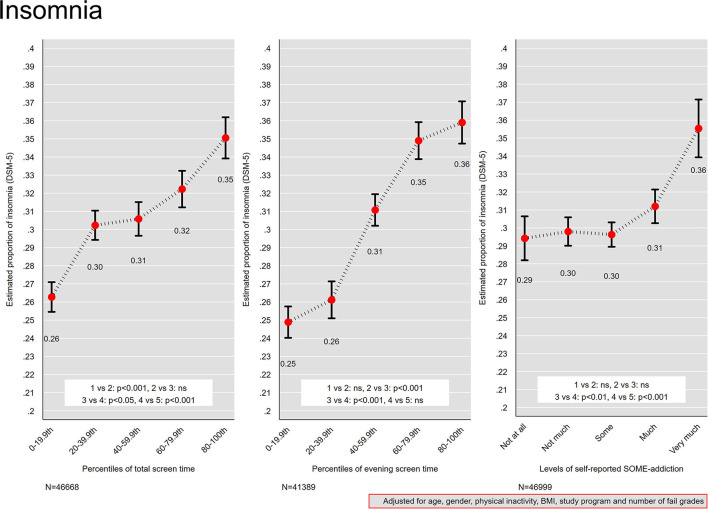
Insomnia. Proportion of insomnia across quintiles of total screen time and evening screen time, and levels of self-reported social media addiction. Results from logistic regression models adjusted for age, gender, physical activity, and body mass index. Bars denote 95 % confidence intervals.

Figure 5 presents the association between screen time and insomnia. For percentiles of total screen time, percentile groups between 20th−59.9th were comparable in relation to insomnia, while an increase from 31 to 32 % was observed between the 40–59.9th group (median screen time of 8 h) and the 60–79th group (median screen time of 10 h). The lowest percentile group (median screen time of 3 h) had the lowest estimated proportion of insomnia (26 %), while the highest proportion (35 %) was observed on the other extreme of the percentile groups (median screen time 12 h). For percentiles of evening screen time, a monotonic association was observed for the four first percentile groups (ranging between 0–79.9th percentile), with a relatively sharp increase in proportion of insomnia across the following groups: 20–39.9th, 40–59.9th and 60–79.9th. The proportion of insomnia rose from 25 % in the 0–19th percentile group (median evening screen time of 7 min) to 36 % in the 80–100th percentile group (median evening screen time 2 h). No differences were observed between the 0–19th and 20–39th percentile groups, or the 60–79.9th and the 80–100th percentile group.

### Social Media Addiction and Insomnia

For levels of self-reported SOME-addiction, the groups “not at all,” “not much” and “some” had the lowest estimated proportion of insomnia (30 %; Figure 5). A higher proportion of insomnia was observed in the “much” group compared to the “some” group (31 vs. 30%), while the “very much” group had significantly higher proportion insomnia compared to the other groups (36 %).

## Discussion

Using data from a national health survey of all Norwegian college and university students, the present study provides evidence of a strong negative association between time spent on screen-based activities and sleep, and where screen use in bed had more consistent negative associations with sleep. These findings suggest a significant role of screen use in relation to students' sleep quantity and quality, and are in line with previous studies on students and young adults (14, 15, 24). The results also demonstrate a central role of social media addiction, with higher rates of insomnia among those with higher levels of self-reported addiction, and a curvilinear relationship with sleep duration, sleep onset latency, and sleep efficiency. There were minimal differences between the fully adjusted associations and the associations adjusted for only age and gender.

The present results show that increasing evening screen time are associated with larger decreases of both sleep quantity and quality, compared to increasing total screen time. Thus, the association between screen time and sleep may largely be driven by evening screen time, as suggested by Christensen et al. (13). It would be of interest to assess the sleep of people exclusively using screens during the day; however, too few participants in the present study did not use screens in bed. Nevertheless, the 0–19th percentile group of evening screen use had a median evening screen time of only 7 min, and also the most favorable sleep outcomes, suggesting that as little evening screen time as possible is the most optimal to promote good sleep. However, the cross-sectional design of the study prohibits drawing conclusions about cause-and-effect, and some studies have indicated that screen use may not directly lead to poor sleep. For example, in a 3-year longitudinal study, Tavernier & Willoughby (41) found that sleep problems predicted more social media use and TV/computer use, and not vice versa. Another study used experience sampling and found that bedtime social media use did not predict poorer sleep the same night (42). These studies hint that screen use may be a way of coping with sleep problems, rather than causing sleep problems. As such, more longitudinal studies assessing the direction of effects are warranted.

In terms of the relationship between social media addiction and sleep, there were U-shaped associations where the responses of “some” addiction was associated with the longest sleep duration, shortest sleep onset latency, and highest sleep efficiency. A key motivation for using social media is social interaction (43), and feeling addicted to social media may in part reflect one's desire to stay in touch with friends. It may be that reporting “some” addiction reflects being socially well-adapted, since so much of young people's social lives happen on social media. One may speculate that indicating no social media addiction reflects social marginalization, in terms of not participating in an important social arena for young people. Conversely, indicating very much addiction may reflect a problematic relationship to social media, where one's preoccupation with social media infringes on other important activities, such as sleep. The U-shaped association was not seen for insomnia, where the “not at all,” “not much,” and “some” addiction groups had similar insomnia rates. This may be related to the nature of the insomnia measure, which is based on sleep problems and not on time-based estimates of sleep, resulting in different associations. Importantly, there were increasing rates of insomnia in the “much” and “very much” groups.

The results also suggest that social media addiction is related to increased screen use in bed, in line with the findings by Rosen et al. (24) and Tandon et al. (25). However, the increase in evening screen time with higher social media addiction was modest, where the difference in median evening screen time between the “not at all” group and the “very much” group was only 13 min. Considering that the difference between those with the lowest and highest evening screen time (0–19th vs. 80–100th percentile groups) was 1 h 54 min, other factors are likely to influence evening screen time beyond social media addiction. Nevertheless, “checking social media” was the most common bedtime activity among both males and females, suggesting that social media use is a highly relevant behavior to consider when studying students' sleep.

In line with previous studies (15, 29, 32), we found gender differences in sleep, screen time, and social media addiction, suggesting that studies of sleep and screen/social media use should account for gender.

### Clinical Relevance of the Results

The present results showed a progressive worsening in sleep efficiency with increasing total screen time, however, sleep efficiency was above 85 % even in the group with a median of 12 h total screen time. A sleep efficiency of 85 % is often used as a lower cut-off for defining clinical sleep problems (44). For evening screen time, on the other hand, the 60–79th percentile group (median evening screen time of 50 min) had a mean sleep efficiency of 85 %, and the 80–100th percentile group (median evening screen time of 2 h) had a mean sleep efficiency of 83 %, suggesting that evening screen time of more than 50 min is associated with clinically impaired sleep efficiency.

All screen time percentile groups had a mean sleep duration between 7 and 8 h. Below 6 has been associated with negative health outcomes (5), and 7–9 h is recommended (3). It is possible that students, who often do not have to attend lectures until late in the morning, are able to sleep in during the week, and are thus able to compensate if they have not slept well (32). Other populations may have stricter working/school hours and consequently their sleep duration is affected more severely by difficulties falling asleep and poor sleep quality. In line with this, Hysing et al. (32) showed that sleep duration increases from adolescence to young adulthood, alongside an increase in the rate of insomnia. Regardless of satisfactory sleep duration, a low sleep efficiency indicates that the person spends a considerable amount of time in bed trying to sleep, which reflects a core problem for those suffering from insomnia (45). The proportion of insomnia rose from 25 to 36 % across groups with increasing evening screen time, attesting to the clinical relevance of this behavior.

### Strengths and Limitations

A strength of the current study was the use of a large and heterogeneous sample. Further, measuring both time-based outcomes (sleep duration, SOL, SE) and insomnia operationalized according to DSM-5 criteria, is a strength of the study, although we did not use other widely used tools such as the Insomnia Severity Index or the Pittsburgh Sleep Quality Index. Another strength is that we adjusted for physical inactivity, being overweight, study program, and number of fail grades in addition to age and gender.

The most important limitation of the study is its cross-sectional design, precluding conclusions about cause and effect. Screen time may interrupt sleep, but individuals who struggle with falling asleep may simply spend more time on screen-based activities than their sleeping counterparts. There may also be a reciprocal relationship; people who are unable to fall asleep at night may engage in screen-based activities, further disrupting sleep (46). We have employed an overall screen-time measure, and have not investigated possible differences across different types of technology and activities. Fossum et al. (15) showed that computer and mobile phone usage was positively associated with insomnia, but that using a gaming console, tablet, watching television, or using an audio player was not. It would also be of interest to investigate specific screen-based activities (e.g., chatting with friends, reading the news, playing games, scrolling through content) in relation to sleep. For example, Kingsbury et al. (47) demonstrated that messaging friends and other active social private use was associated with decreased odds of self-injury and suicidal behaviors, while active social public use (e.g., status updates) and also social comparison was associated with increased odds for these behaviors.

Another limitation is that the assessment of sleep and screen use was based on self-report. Healthy adults have been shown to over-report their total sleep time (48, 49) and people with insomnia have been shown to under-report their total sleep time. Moreover, it is unknown whether people can accurately estimate their screen time (50). One study found that people tend to overestimate how much time they spend on social media, but underestimate how many times they visit social media each day, and that heavy users and young people provide more inaccurate estimates (51). Future studies could benefit from using objective measures of screen time, for example by extracting user data from participants' smartphones and other screen-based devices. Furthermore, unmeasured confounding may have impacted the results.

Lastly, measuring addiction using only one item may not have been optimal to capture addictive social media use among the participants, and this may explain the modest association with screen time and sleep. Rather, indicating that one is addicted may reflect other characteristics such as personality traits or response styles. We did not measure the different core concepts of addiction (52), and thus the present finding of an association between social media addiction and sleep should be regarded a preliminary indication of a role of social media addiction in screen time and sleep, and caution is necessary when generalizing the findings. Future studies should use more elaborate measures of addictive behaviors and explore other motivations or mechanisms influencing screen time and sleep.

## Conclusion

The present findings suggest that screen use plays an important role in students' sleep quantity and quality, where evening screen time has a stronger relationship with sleep compared to total daily screen time. Sleep problems are pervasive among students, and reducing evening screen use may contribute to improved sleep, although further evidence is needed to establish cause-and-effect. The results also suggest a role of social media addiction, and addictive social media use may be a target for intervention in order to reduce evening screen time.

## Data Availability Statement

The data analyzed in this study is subject to the following licenses/restrictions: Data will be made available upon reasonable request. Requests to access these datasets should be directed to Gunnhildjohnsen.Hjetland@fhi.no.

## Ethics Statement

The SHoT2018 study was reviewed and approved by Regional Committee for Medical and Health Ethics in Western Norway (No. 2017/1176). The patients/participants provided their written informed consent to participate in this study.

## Author Contributions

GH: conceptualization, writing—original draft preparation, and writing—review and editing. JS: conceptualization, methodology, formal analysis, writing—original draft preparation, writing—review and editing, and visualization. MH: investigation and writing—review and editing. BS: conceptualization, investigation, writing—original draft preparation, and writing—review and editing. All authors have read and approved the manuscript for submission.

## Funding

The SHoT2018 study has received funding from the Norwegian Ministry of Education and Research (2017) and the Norwegian Ministry of Health and Care Services (2016). The funding sources had no involvement in the study design or the collection, analysis, and interpretation of data, in the writing of the report, or in the decision to submit the article for publication.

## Conflict of Interest

The authors declare that the research was conducted in the absence of any commercial or financial relationships that could be construed as a potential conflict of interest.

## Publisher's Note

All claims expressed in this article are solely those of the authors and do not necessarily represent those of their affiliated organizations, or those of the publisher, the editors and the reviewers. Any product that may be evaluated in this article, or claim that may be made by its manufacturer, is not guaranteed or endorsed by the publisher.

## References

[1] Becker SP Jarrett MA Luebbe AM Garner AA Burns GL Kofler MJ. Sleep in a large, multi-university sample of college students: sleep problem prevalence, sex differences, and mental health correlates. Sleep Health. (2018) 4:174–81. 10.1016/j.sleh.2018.01.001 29555131 PMC5863586

[2] Lund HG Reider BD Whiting AB Prichard JR. Sleep patterns and predictors of disturbed sleep in a large population of college students. J Adolesc Health. (2010) 46:124–32. 10.1016/j.jadohealth.2009.06.016 20113918

[3] Hirshkowitz M Whiton K Albert SM Alessi C Bruni O DonCarlos L. National Sleep Foundation's updated sleep duration recommendations. Sleep Health. (2015) 1:233–43. 10.1016/j.sleh.2015.10.004 29073398

[4] Sivertsen B Vedaa Ø Harvey AG Glozier N Pallesen S Aarø LE. Sleep patterns and insomnia in young adults: A national survey of Norwegian university students. J Sleep Res. (2019) 28:e12790. 10.1111/jsr.12790 30515935

[5] Itani O Jike M Watanabe N Kaneita Y. Short sleep duration and health outcomes: a systematic review, meta-analysis, and meta-regression. Sleep Med. (2017) 32:246–56. 10.1016/j.sleep.2016.08.006 27743803

[6] Sivertsen B Hysing M Harvey AG Petrie KJ. The epidemiology of insomnia and sleep duration across mental and physical health: the SHoT study. Front Psychol. (2021) 12:2309. 10.3389/fpsyg.2021.662572 34194368 PMC8236531

[7] Tobaldini E Costantino G Solbiati M Cogliati C Kara T Nobili L. Sleep, sleep deprivation, autonomic nervous system and cardiovascular diseases. Neurosci Biobehav Rev. (2017) 74:321–9. 10.1016/j.neubiorev.2016.07.004 27397854

[8] Blake MJ Trinder JA Allen NB. Mechanisms underlying the association between insomnia, anxiety, and depression in adolescence: implications for behavioral sleep interventions. Clin Psychol Rev. (2018) 63:25–40. 10.1016/j.cpr.2018.05.006 29879564

[9] Danielsson NS Harvey AG MacDonald S Jansson-Fröjmark M Linton SJ. Sleep disturbance and depressive symptoms in adolescence: the role of catastrophic worry. J Youth Adolesc. (2013) 42:1223–33. 10.1007/s10964-012-9811-6 22968332

[10] Curcio G Ferrara M De Gennaro L. Sleep loss, learning capacity and academic performance. Sleep Med Rev. (2006) 10:323–37. 10.1016/j.smrv.2005.11.001 16564189

[11] Hartmann ME Prichard JR. Calculating the contribution of sleep problems to undergraduates' academic success. Sleep Health. (2018) 4:463–71. 10.1016/j.sleh.2018.07.002 30241662

[12] Okano K Kaczmarzyk JR Dave N Gabrieli JD Grossman JC. Sleep quality, duration, and consistency are associated with better academic performance in college students. NPJ science of learning. (2019) 4:1–5. 10.1038/s41539-019-0055-z 31583118 PMC6773696

[13] Christensen MA Bettencourt L Kaye L Moturu ST Nguyen KT Olgin JE. Direct measurements of smartphone screen-time: relationships with demographics and sleep. PLoS ONE. (2016) 11:e0165331. 10.1371/journal.pone.0165331 27829040 PMC5102460

[14] Levenson JC Shensa A Sidani JE Colditz JB Primack BA. The association between social media use and sleep disturbance among young adults. Prevent Med. (2016) 85:36–41. 10.1016/j.ypmed.2016.01.001 26791323 PMC4857587

[15] Fossum IN Nordnes LT Storemark SS Bjorvatn B Pallesen S. The association between use of electronic media in bed before going to sleep and insomnia symptoms, daytime sleepiness, morningness, and chronotype. Behav Sleep Med. (2014) 12:343–57. 10.1080/15402002.2013.819468 24156294

[16] Lastella M Rigney G Browne M Sargent C. Electronic device use in bed reduces sleep duration and quality in adults. Sleep Biol Rhythms. (2020) 18:121–9. 10.1007/s41105-019-00251-y

[17] Cain N Gradisar M. Electronic media use and sleep in school-aged children and adolescents: A review. Sleep Med. (2010) 11:735–42. 10.1016/j.sleep.2010.02.006 20673649

[18] Scott H Woods HC. Fear of missing out and sleep: Cognitive behavioural factors in adolescents' nighttime social media use. J Adolesc. (2018) 68:61–5. 10.1016/j.adolescence.2018.07.009 30031979

[19] Dijk D-J Cajochen C. Melatonin and the circadian regulation of sleep initiation, consolidation, structure, and the sleep EEG. J Biol Rhythms. (1997) 12:627–35. 10.1177/074873049701200618 9406038

[20] Grønli J Byrkjedal IK Bjorvatn B Nødtvedt Ø Hamre B Pallesen S. Reading from an iPad or from a book in bed: the impact on human sleep: a randomized controlled crossover trial. Sleep Med. (2016) 21:86–92. 10.1016/j.sleep.2016.02.006 27448477

[21] Harbard E Allen NB Trinder J Bei B. What's keeping teenagers up? prebedtime behaviors and actigraphy-assessed sleep over school and vacation. J Adolesc Health. (2016) 58:426–32. 10.1016/j.jadohealth.2015.12.011 26874590

[22] Kietzmann JH Hermkens K McCarthy IP Silvestre BS. Social media? get serious! understanding the functional building blocks of social media. Bus Horiz. (2011) 54:241–51. 10.1016/j.bushor.2011.01.005

[23] Kuss DJ Griffiths MD. Social networking sites and addiction: Ten lessons learned. Int J Environ Res Public Health. (2017) 14:311. 10.3390/ijerph14030311 28304359 PMC5369147

[24] Rosen L Carrier LM Miller A Rokkum J Ruiz A. Sleeping with technology: cognitive, affective, and technology usage predictors of sleep problems among college students. Sleep Health. (2016) 2:49–56. 10.1016/j.sleh.2015.11.003 29073453

[25] Tandon A Kaur P Dhir A Mäntymäki M. Sleepless due to social media? investigating problematic sleep due to social media and social media sleep hygiene. Comput Hum Behav. (2020) 113:106487. 10.1016/j.chb.2020.106487

[26] Wolniczak I Cáceres-DelAguila JA Palma-Ardiles G Arroyo KJ Solís-Visscher R Paredes-Yauri S. Association between Facebook dependence and poor sleep quality: a study in a sample of undergraduate students in Peru. PLoS ONE. (2013) 8:e59087. 10.1371/journal.pone.0059087 23554978 PMC3595202

[27] Xanidis N Brignell CM. The association between the use of social network sites, sleep quality and cognitive function during the day. Comput Human Behav. (2016) 55:121–6. 10.1016/j.chb.2015.09.004

[28] Panova T Carbonell X. Is smartphone addiction really an addiction? J Behav Addict. (2018) 7:252–9. 10.1556/2006.7.2018.49 29895183 PMC6174603

[29] Andreassen CS Torsheim T Brunborg GS Pallesen S. Development of a Facebook addiction scale. Psychol Rep. (2012) 110:501–17. 10.2466/02.09.18.PR0.110.2.501-517 22662404

[30] Hale L Guan S. Screen time and sleep among school-aged children and adolescents: a systematic literature review. Sleep Med Rev. (2015) 21:50–8. 10.1016/j.smrv.2014.07.007 25193149 PMC4437561

[31] Birkjær M Kaats M. Does social media really pose a threat to young people's well-being? (2019). Available Online at: https://www.norden.org/en/publication/does-social-media-really-pose-threat-young-peoples-well-being-0

[32] Hysing M Harvey AG Bøe T Heradstveit O Vedaa Ø Sivertsen B. Trajectories of sleep problems from adolescence to adulthood. linking two population-based studies from Norway. Sleep Med. (2020) 75:411–7. 10.1016/j.sleep.2020.08.035 32971382

[33] Christofaro DGD De Andrade SM Mesas AE Fernandes RA Farias Junior JC. Higher screen time is associated with overweight, poor dietary habits and physical inactivity in Brazilian adolescents, mainly among girls. Eur J Sport Sci. (2016) 16:498–506. 10.1080/17461391.2015.1068868 26239965

[34] Delfino LD dos Santos Silva DA Tebar WR Zanuto EF Codogno JS Fernandes RA. Screen time by different devices in adolescents: association with physical inactivity domains and eating habits. J Sports Med Phys Fitness. (2017) 58:318–25. 10.23736/S0022-4707.17.06980-8 28462567

[35] Hasler G Buysse DJ Klaghofer R Gamma A Ajdacic V Eich D. The association between short sleep duration and obesity in young adults: a 13-year prospective study. Sleep. (2004) 27:661–6. 10.1093/sleep/27.4.661 15283000

[36] Melkevik O Torsheim T Iannotti RJ Wold B. Is spending time in screen-based sedentary behaviors associated with less physical activity: a cross national investigation. Int J Behav Nutri Physic Activ. (2010) 7:1–10. 10.1186/1479-5868-7-46 20492643 PMC3224890

[37] Sivertsen B Råkil H Munkvik E Lønning KJ. Cohort profile: the SHoT-study, a national health and well-being survey of Norwegian university students. BMJ Open. (2019) 9:e025200. 10.1136/bmjopen-2018-025200 30670525 PMC6347864

[38] Hysing M Pallesen S Stormark KM Jakobsen R Lundervold AJ Sivertsen B. Sleep and use of electronic devices in adolescence: results from a large population-based study. BMJ Open. (2015) 5:e006748. 10.1136/bmjopen-2014-006748 25643702 PMC4316480

[39] Grasdalsmoen M Eriksen HR Lønning KJ Sivertsen B. Physical exercise and body-mass index in young adults: a national survey of Norwegian university students. BMC Public Health. (2019) 19:1–9. 10.1186/s12889-019-7650-z 31646998 PMC6813074

[40] World Health Organization. Physical status: The Use and Interpretation of Anthropometry. Report of a WHO Expert Commitee. WHO Techical Report Series (1995). Available online at: https://apps.who.int/iris/bitstream/handle/10665/37003/W?sequence=1 8594834

[41] Tavernier R Willoughby T. Sleep problems: predictor or outcome of media use among emerging adults at university? J Sleep Res. (2014) 23:389–96. 10.1111/jsr.12132 24552437

[42] Das-Friebel A Lenneis A Realo A Sanborn A Tang NK Wolke D. Bedtime social media use, sleep, and affective wellbeing in young adults: an experience sampling study. J Child Psychol Psychiatry. (2020) 61:1138–49. 10.1111/jcpp.13326 32924153

[43] Kuss DJ Griffiths MD. Online social networking and addiction—a review of the psychological literature. Int J Environ Res Public Health. (2011) 8:3528–52. 10.3390/ijerph8093528 22016701 PMC3194102

[44] Lacks P Morin CM. Recent advances in the assessment and treatment of insomnia. J Consult Clin Psychol. (1992) 60:586. 10.1037/0022-006X.60.4.586 1506506

[45] Morin CM Benca R. Chronic insomnia. Lancet. (2012) 379:1129–41. 10.1016/S0140-6736(11)60750-2 22265700

[46] Liu S Wing YK Hao Y Li W Zhang J Zhang B. The associations of long-time mobile phone use with sleep disturbances and mental distress in technical college students: a prospective cohort study. Sleep. (2019). 42:zsy213. 10.1093/sleep/zsy213 30395300

[47] Kingsbury M Reme B-A Skogen JC Sivertsen B Øverland S Cantor N. Differential associations between types of social media use and university students' non-suicidal self-injury and suicidal behavior. Comput Human Behav. (2021) 115:106614. 10.1016/j.chb.2020.106614

[48] Matthews KA Patel SR Pantesco EJ Buysse DJ Kamarck TW Lee L. Similarities and differences in estimates of sleep duration by polysomnography, actigraphy, diary, and self-reported habitual sleep in a community sample. Sleep Health. (2018) 4:96–103. 10.1016/j.sleh.2017.10.011 29332687 PMC5771411

[49] Lauderdale DS Knutson KL Yan LL Liu K Rathouz PJ. Self-reported and measured sleep duration: how similar are they? Epidemiology. (2008) 8:838–845. 10.1097/EDE.0b013e318187a7b0 18854708 PMC2785092

[50] Kaye LK Orben A Ellis DA Hunter SC Houghton S. The conceptual and methodological mayhem of “screen time.” *Int J Environ Res Public Health*. (2020) 17:3661. 10.3390/ijerph17103661 32456054 PMC7277381

[51] Ernala SK Burke M Leavitt A Ellison NB. “How well do people report time spent on Facebook? An evaluation of established survey questions with recommendations,” In:. Proceedings of the. (2020). CHI Conference on Human Factors in Computing Systems: Honolulu. (2020).

[52] Griffiths M. A ‘components' model of addiction within a biopsychosocial framework. J Subst Use. (2005) 10:191–7. 10.1080/14659890500114359

